# Cardiovascular magnetic resonance feature-tracking assessment of myocardial mechanics: Intervendor agreement and considerations regarding reproducibility

**DOI:** 10.1016/j.crad.2015.05.006

**Published:** 2015-09

**Authors:** A. Schuster, V.-C. Stahnke, C. Unterberg-Buchwald, J.T. Kowallick, P. Lamata, M. Steinmetz, S. Kutty, M. Fasshauer, W. Staab, J.M. Sohns, B. Bigalke, C. Ritter, G. Hasenfuß, P. Beerbaum, J. Lotz

**Affiliations:** a Department of Cardiology and Pneumology, Georg-August University, Göttingen, Germany; b DZHK (German Centre for Cardiovascular Research), Germany; c Division of Imaging Sciences and Biomedical Engineering, The Rayne Institute, St. Thomas' Hospital, King's College London, London, UK; d Institute for Diagnostic and Interventional Radiology, Georg-August University, Göttingen, Germany; e Department of Computer Science, University of Oxford, Oxford, UK; f Department of Paediatric Cardiology and Intensive Care Medicine, Georg-August-University Göttingen, Göttingen, Germany; g Children's Hospital and Medical Center Joint Division of Pediatric Cardiology, University of Nebraska / Creighton University, Omaha, NE, USA; h Department of Cardiology, Campus Benjamin Franklin, Charité Berlin, Hindenburgdamm 30, 12200 Berlin, Germany; i Department of Paediatric Cardiology, Hannover Medical School, Hannover, Germany

## Abstract

**Aim:**

To assess intervendor agreement of cardiovascular magnetic resonance feature tracking (CMR-FT) and to study the impact of repeated measures on reproducibility.

**Materials and methods:**

Ten healthy volunteers underwent cine imaging in short-axis orientation at rest and with dobutamine stimulation (10 and 20 μg/kg/min). All images were analysed three times using two types of software (TomTec, Unterschleissheim, Germany and Circle, cvi^42^, Calgary, Canada) to assess global left ventricular circumferential (Ecc) and radial (Err) strains and torsion. Differences in intra- and interobserver variability within and between software types were assessed based on single and averaged measurements (two and three repetitions with subsequent averaging of results, respectively) as determined by Bland–Altman analysis, intraclass correlation coefficients (ICC), and coefficient of variation (CoV).

**Results:**

Myocardial strains and torsion significantly increased on dobutamine stimulation with both types of software (*p<*0.05). Resting Ecc and torsion as well as Ecc values during dobutamine stimulation were lower measured with Circle (*p<*0.05). Intra- and interobserver variability between software types was lowest for Ecc (ICC 0.81 [0.63–0.91], 0.87 [0.72–0.94] and CoV 12.47% and 14.3%, respectively) irrespective of the number of analysis repetitions. Err and torsion showed higher variability that markedly improved for torsion with repeated analyses and to a lesser extent for Err. On an intravendor level TomTec showed better reproducibility for Ecc and torsion and Circle for Err.

**Conclusions:**

CMR-FT strain and torsion measurements are subject to considerable intervendor variability, which can be reduced using three analysis repetitions. For both vendors, Ecc qualifies as the most robust parameter with the best agreement, albeit lower Ecc values obtained using Circle, and warrants further investigation of incremental clinical merit.

## Highlights

• This is the first comparison of two types of CMR-FT software resulting in clinically valuable inter-vendor agreement data.
• Assessment of myocardial strain and torsion is feasible with both types of software at rest and with dobutamine stimulation.
• For both vendors, Ecc qualifies as the most robust parameter with the lowest variability.

## Introduction

Heart failure is characterised by high mortality irrespective of the predominance of either systolic or diastolic functional impairment.^1^, ^2^ Several imaging techniques are available to characterise its aetiology and severity, amongst which cardiovascular magnetic resonance (CMR) has a pivotal role.^3^, ^4^, ^5^ In particular, the opportunity of easy and fast quantitative image analyses makes this technique attractive.^6^ There is evidence to suggest that quantitative deformation imaging derived strain assessment based on echocardiographic speckle tracking has higher value for the prediction of mortality than ejection fraction (EF) in consecutive patients subjected to echocardiography.^7^ CMR-derived myocardial feature tracking (FT), a technique analogous to echocardiography speckle tracking, derives similar quantitative deformation parameters from routinely available steady state free precession (SSFP) cine sequences. Reasonable agreement between speckle tracking and CMR-FT has been demonstrated.^8^ Furthermore, CMR-FT agrees well with myocardial tagging,^9^ which is considered the reference standard for CMR quantitative wall-motion assessment, but the former does not require the acquisition of additional sequences.^10^ Its clinical applicability has been demonstrated in a variety of cardiovascular diseases,^8^, ^11^, ^12^, ^13^ its feasibility of detailed assessments of systolic and diastolic cardiovascular physiology has been demonstrated,^14^, ^15^ and there is evidence of prognostic relevance in dilated cardiomyopathy.^13^ Although the vast majority of such studies have been carried out with the software provided by TomTec Imaging Systems (Diogenes or 2D Cardiac Performance Analysis-MR, TomTec GmbH, Unterschleissheim, Germany)^16^ recently Circle Cardiovascular Imaging (cvi^42^, Calgary, Canada) have introduced an alternative tool called Tissue Tracking. Given the fact that a widespread clinical use of these new measures of deformation is highly desirable and likely important, prerequisites to achieve this goal are to ensure that the assessments are reproducible and comparable with a high amount of intervendor agreement. Therefore, the aim of the present study was to assess the reproducibility and intervendor agreement of both commercially available types of software for the derivation of ventricular circumferential (Ecc) and radial (Err) strains, as well as rotational mechanics expressed as left ventricular (LV) torsion.

## Material and methods

The study cohort consisted of 10 healthy volunteers. CMR imaging was carried out on a 1.5 T system (Intera R 12.6.1.3, Philips Medical Systems, Best, The Netherlands). All participants gave written informed consent after approval of the study protocol by the Institutional Review Board at the University of Nebraska Medical Center.

### CMR imaging

The CMR examination was carried out in the supine position using a five-channel cardiac surface coil. Electrocardiogram (ECG)-gated SSFP cine sequences were acquired during brief periods of breath-holding in 12 to 14 equidistant short-axis planes completely covering the LV. Typical CMR parameters were as follows: 8 mm section thickness; 1–2 mm gap; 360×480 mm field of view; 196×172 matrix size. Dobutamine stress CMR imaging was performed as previously described.^17^ Complete short-axis stacks were acquired at rest and with 10 and 20 μg/kg/min dobutamine, respectively.

### CMR-FT

CMR-FT was performed using dedicated software provided by TomTec Imaging Systems (2D CPA MR, Cardiac Performance Analysis, Version 1.1.2.36) and Circle Cardiovascular Imaging (Tissue Tracking, cvi^42^). For the purposes of this paper the different software tools are referred to as “TomTec” and “Circle”. Identical short axis sections were analysed at apical, mid-ventricular, and basal levels to compare short-axis-derived global LV Ecc and Err (based on all three analysed sections) alongside the time-to-peak (TPK) strain duration. Short-axis CMR images were analysed at rest and with 10 and 20 μg/kg/min dobutamine, respectively. Myocardial torsion was calculated from the rotational raw data provided with the TomTec software using an in-house-developed post-processing tool as recently described by the authors' group.^15^ The model underlying this assessment makes use of linear interpolation and takes standardized rotational measurements at 25 and 75% LV locations after the analysis of a whole LV short axis stack. In this model the most apical section showing LV cavity at end-systole is considered at the 0% LV location and the most basal section including a complete circumference of myocardium at end-systole is considered at the 100% LV location. In comparison to TomTec, Circle commercially provides torsion measurements within its software interface. This is done by manually choosing an apical and basal section. In order to allow accurate comparisons between vendors, apical and basal sections at the closest distance to 25% and 75% LV locations were chosen.

With both types of software LV endocardial and epicardial borders were manually delineated in all analysed sections with the initial contour set at end-diastole. In case of insufficient tracking, as defined by apparent deviations of the contours from the endocardial and epicardial borders, contours were manually corrected and the algorithm reapplied. The tracking was repeated three times in all sections. One single observer analysed all data using both types of software. Intra-observer variability was derived from the repetition of the analysis after 4 weeks. The analysis of a second skilled observer for both types of software was used to assess interobserver reproducibility.

Reported results are based on the average of three analysis repetitions (R3). To study the impact of repeated measurements on reproducibility, the reproducibility derived from results based on a single repetition (R1), averaged results for two (R2) and three repetitions (R3) were compared with each other.

### Statistical analysis

Statistical analysis was conducted using Microsoft Excel and IBM SPSS Statistics version 22 for Windows. Data are expressed as mean (± standard deviations). Pairwise non-parametric data at rest and with increasing levels of dobutamine were compared using the Wilcoxon test. Significance was determined at <0.05. The intra- and interobserver variability was assessed using three different methods: intraclass correlation coefficients (ICC), Bland–Altman analysis,^18^ and coefficients of variation (CoV). The CoV was defined as the standard deviation of the differences divided by the mean.^19^ The level of agreement was defined as previously described: excellent for ICC>0.74, good for ICC = 0.60–0.74, fair for ICC = 0.40–0.59, and poor for ICC<0.4.^20^

## Results

**Figure 1:**
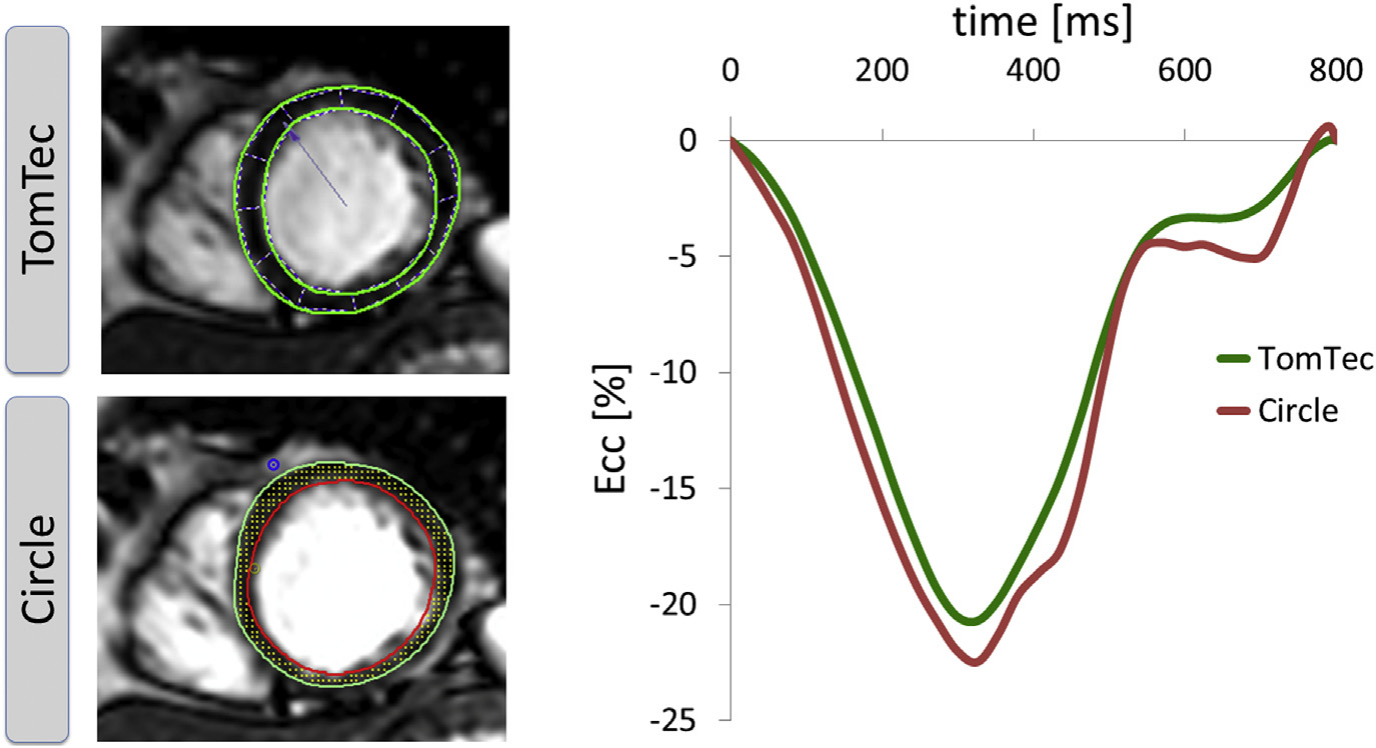
Example of the derivation of LV Ecc curves, using the two commercially available CMR-FT software types.

Demographics are displayed in Table 1. Quantitative analysis was performed in all subjects. Fig 1 shows a representative example of the derivation of Ecc with both types of software, respectively. Although all scans were successfully analysed using TomTec (100%) one volunteer was excluded with intermediate dose dobutamine stimulation (20 μg/kg/min) from the Circle group due to insufficient border tracking (97% success rate in total). The time for repeat-analysis (three repetitions) of a given case (when only considering the analysis of three sections with both types of software) including the tracking at rest and with the respective dobutamine levels did not vary between the different types of software and took 27–35 minutes on average. Conversely, the analysis time based on a single repetition only, took 9–12 minutes with either type of software.

### Quantification of myocardial strain

**Figure 2:**
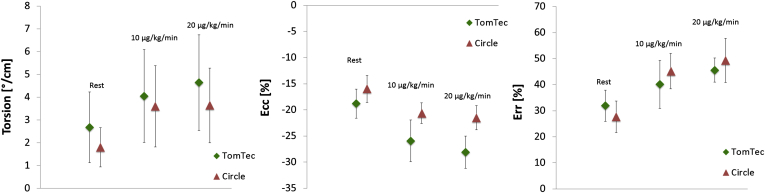
Changes in the Ecc and Err LV strain and myocardial torsion during dobutamine stimulation: The figure shows changes in myocardial torsion (left panel), Ecc (middle panel), and Err (right panel) at rest and with dobutamine stimulation (10 and 20 μg/kg/min).

The changes of myocardial strain in response to dobutamine stimulation are illustrated in Fig 2. There was a significant increase in Ecc and TPK Ecc at both levels of dobutamine (10 and 20 μg/kg/min; *p*<0.05) using TomTec (Table 2). Similarly, with Circle the Ecc and TPK Ecc significantly increased from rest to 10 and 20 μg/kg/min of dobutamine, respectively (*p*<0.05; Table 2). There was no significant increase from 10 to 20 μg/kg/min of dobutamine (*p*=0.374; Table 2). There were significantly lower Ecc values derived from Circle as compared to TomTec at rest (*p*<0.05) and with 10 (*p*<0.05) and 20 μg/kg/min of dobutamine (*p*<0.05; Table 2).

Err significantly increased from rest to 20 and between 10 and 20 μg/kg/min of dobutamine using Tom Tec (*p*<0.05; Table 2). The corresponding TPK Err significantly increased from rest to 10 and 20 μg/kg/min of dobutamine (*p*<0.05) but not between 10 and 20 μg/kg/min of dobutamine (*p*=0.125; Table 2). With Circle Err and TPK Err significantly increased from rest to 10 and to 20 μg/kg/min of dobutamine, respectively (*p*<0.05; Table 2). There was no significant increase from 10 to 20 μg/kg/min of dobutamine (*p*=0.139; *p*=0.051, for Err and TPK Err respectively; Table 2) and no significant difference in Err values derived from either software type (*p*>0.05 for all parameters). There were significantly increased strain values with dobutamine stress as compared to rest irrespective of the number of analysis repetitions (*p*<0.05, data not shown).

### Quantification of myocardial torsion

A significant increase in myocardial torsion was measured between rest and both levels of dobutamine, but not between 10 and 20 μg/kg/min of dobutamine using either software type (*p*<0.05; Fig 2, Table 2). The change in TPK torsion did not reach statistical significance between rest and 10 μg/kg/min of dobutamine using TomTec (*p*=0.13). All other comparisons reached statistical significance (*p*<0.05) (Table 2). There was significant lower torsion at rest measured with Circle as compared to TomTec (*p*<0.05). There was no significant difference in torsion derived from either software during dobutamine stress (*p*>0.05 for all parameters).

### Intervendor agreement and reproducibility

**Figure 3:**
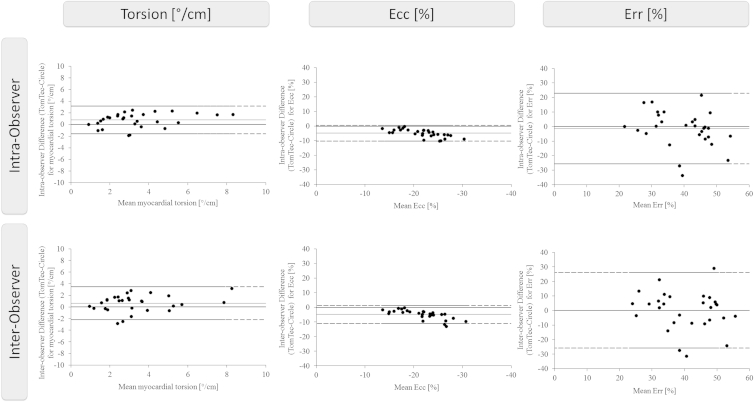
Intervendor agreement of global strain and myocardial torsion as determined by feature tracking. Bland–Altman plots with limits of agreement (95% confidence intervals) demonstrate the intervendor reproducibility of CMR-FT-derived myocardial torsion, global left ventricular Ecc and global left ventricular Err both on the intra-observer and interobserver level. The data shown is based on the measurements at rest and with dobutamine stress. Reproducibility is shown for averaged results based on three repeated measurements (R3).

**Figure 4:**
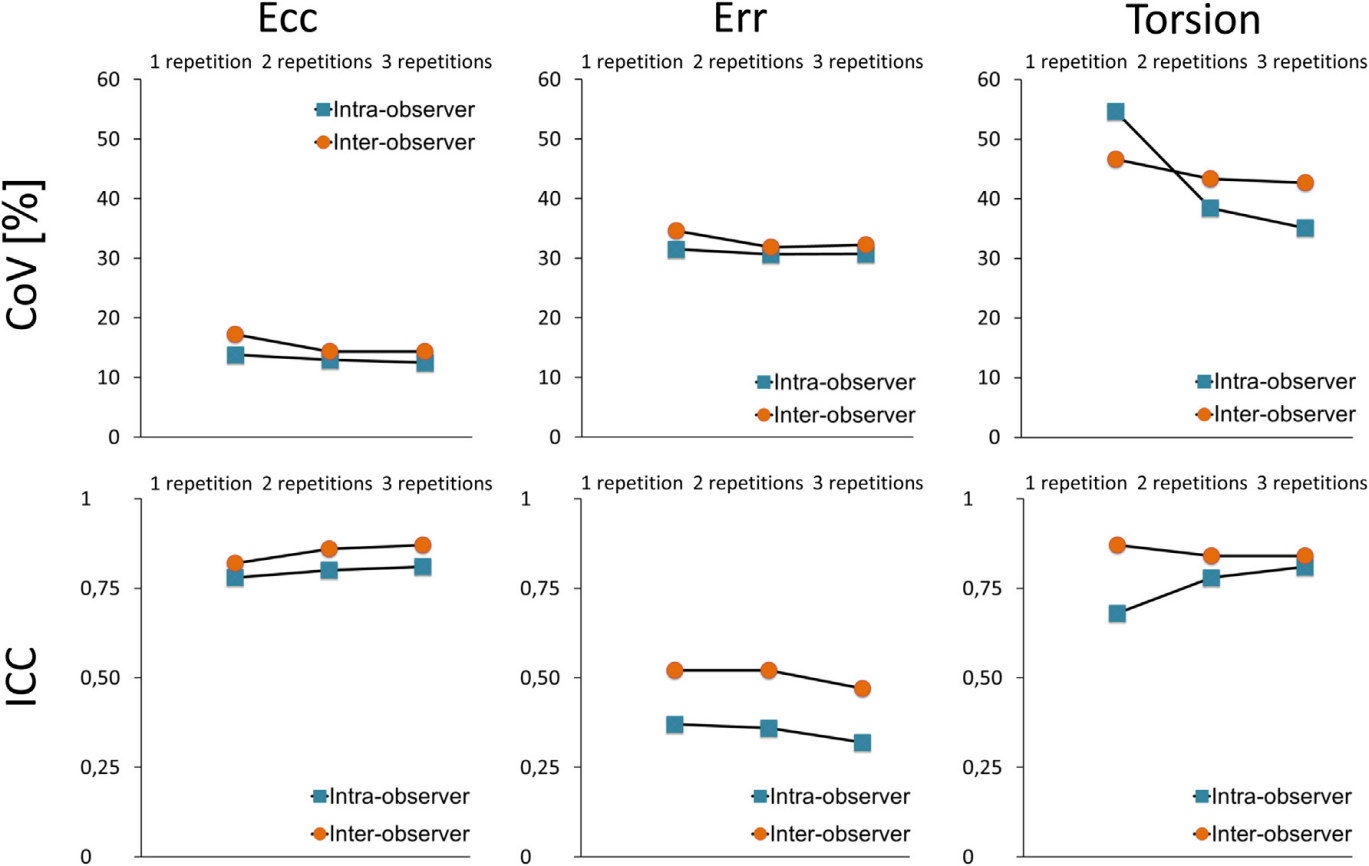
Intervendor agreement of global Err and myocardial torsion as assessed by CoV and ICC. The graphs show the CoV in percent and the ICC on an intra-observer and interobserver level for global left ventricular Ecc, global left ventricular Err, and myocardial torsion. The change in reproducibility (based on CoV and ICC) is shown when deriving the results from a single analysis seen on the left of each graph (‘1 repetition’), averaging of two analyses ‘2 repetitions’ (middle of each graph) and averaging three analysis runs (‘3 repetitions’ right of each graph). The reproducibility results presented in theses graphs are based on strain and torsion values derived at rest and during dobutamine stimulation.

Intervendor variability was lowest for Ecc, and higher for myocardial torsion and Err on an intra- and interobserver level based on three analysis repetitions (R3; Fig 3). Intervendor agreement was generally lower than intravendor agreement for both types of software. Ecc was the least variable parameter for both types of software. Although TomTec showed better reproducibility for Ecc and myocardial torsion as compared to Circle, Circle had better reproducibility than TomTec for Err (Table 3, Fig 4). There was no reduction in intervendor agreement and reproducibility with dobutamine stress (data not shown).

### Impact of repeated measurements on reproducibility

The results based on three repetitions (R3) are shown in Table 3 as compared to results based on two repetitions (R2; Table 4) and to results relying on single analyses (R1; Table 5). The intervendor agreement and the reproducibility within the individual software types of most assessed parameters were improved by repeated measurements both on the intra-observer and interobserver level. Whilst there was relatively little impact on Ecc with intervendor agreement on the intra- (R3: ICC 0.81 [0.63–0.91] CoV 12.47%; R1: ICC 0.78 [0.58–0.9] CoV 13.8%) and interobserver level (R3: ICC 0.87 [0.72–0.94] CoV 14.3%; R1: ICC 0.82 [0.62–0.92] CoV 17.2%) this effect was more pronounced for myocardial torsion on the intra- (R3: ICC 0.81 [0.63–0.9] CoV 35.08%; R1: ICC 0.68 [0.42–0.83] CoV 54.65%) and interobserver level (R3: ICC 0.84 [0.65–0.92] CoV 42.73%; R1: ICC 0.87 [0.72–0.94] CoV 46.65%). There was little impact on the intervendor agreement of Err at the intra- (R3: ICC 0.32 [0–0.61] CoV 30.71%; R1: ICC 0.37 [0.02–0.65] CoV 31.53%) and interobserver level (R3: ICC 0.47 [0–0.75] CoV 32.2%; R1: ICC 0.52 [0–0.77] CoV 34.55%).

## Discussion

To the authors' knowledge, this study is the first comparison of different types of commercially available CMR-FT software, and presents several notable findings. First, intervendor agreement between the two software types is reasonable, with the best agreement for Ecc, and worse but acceptable agreements for myocardial torsion and Err. Second, there is significantly lower Ecc and myocardial torsion at rest and lower Ecc measured with dobutamine stimulation using Circle as compared to TomTec. Third, averaging of the results of repeated analyses increases both intervendor agreement and intravendor reproducibility; however the benefit is relatively low, considering that doubling or tripling of analysis times would be required. Lastly, although both software types show acceptable intravendor reproducibility, it is important to note that the Circle software shows slightly more variability for Ecc and myocardial torsion as compared to TomTec. Conversely, TomTec shows slightly more variability for Err measurements compared to Circle.

Since the introduction of CMR-FT in 2009,^16^ it has found widespread clinical and research applications in various adult and congenital disorders.^8^, ^9^, ^11^, ^12^, ^14^, ^21^ The increased demand for this relatively young technology necessitates the availability of quick and efficient post-processing software. Although, historically, such software has been provided by TomTec, Circle only recently released their version. Nevertheless, results need to be comparable and ideally interchangeable between different types of software to allow widespread clinical use. Within the present study, post-processing times were comparable between the software types making them equally applicable for clinical use. The fact that Ecc and resting myocardial torsion measured with Circle showed significantly lower values as compared to TomTec could potentially limit the interchangeability of results between vendors. Clearly, there is a need to consider these inherent differences when comparing results from either type of software. Notwithstanding these considerations, the underestimation (from a Circle perspective) or overestimation (from a TomTec perspective) in Ecc was consistent in the three experimental conditions and reproducible through the three repetitions, which may allow future work to introduce correction factors to account for these differences. The average difference between vendors was 4.8% (see Table 3) a considerable value compared to the range of strain observed in this population (10 to 30%, see Fig 2). Vendor-induced variability between TomTec and Circle was lowest for Ecc (see Table 3, Table 4, Table 5). The finding of high reproducibility of Ecc is in line with previously published literature.^9^, ^10^, ^11^, ^22^, ^23^ There is evidence from studies that used TomTec suggesting that Ecc is the CMR-FT parameter with the highest reproducibility in health^10^ and disease,^11^ irrespective of field strength.^23^ Furthermore, of all CMR-FT-derived parameters, Ecc has been shown to have the highest interstudy reproducibility^22^ as well as the best agreement with echocardiographical speckle tracking.^8^

Myocardial torsion and Err were subject to higher intervendor variability compared to Ecc and to lower bias between vendors. Although Err showed no significant bias between vendors, there was significant underestimation of torsion at rest, but not during dobutamine stimulation using Circle. The variability associated with torsion may well be explained by the fact that the methodology that has been validated with TomTec makes use of linear interpolation and standardized measurements at predefined anatomical LV locations as compared to Circle that derives rotational mechanics directly from the analysed sections.^15^ Based on ICC, Err showed the lowest intravendor reproducibility and intervendor agreement. Conversely, torsion showed the lowest intravendor reproducibility and intervendor agreement based on CoV.

It is important to note that Ecc and myocardial torsion reached slightly higher intravendor reproducibility with TomTec as compared to Circle. This may be related to the fact that TomTec has been around for several years and been subjected to changes of the tracking algorithm several times (last change in December 2012); however, Circle showed better reproducibility for Err than TomTec.

Considering these results, further refinements in the performance and subsequent increases in agreement between vendors and within vendors seem highly desirable. To achieve this, the impact of repeated measurements and subsequent averaging of results was tested. Although each of the parameters shows somewhat improved intervendor agreement and intravendor reproducibility with repeated analysis runs, this effect is most evident for myocardial torsion (reduction of intervendor CoV from 55% to 38%, see Table 3, Table 5). In comparison, three repetitions have a lower effect on Ecc as opposed to the lesser reproducible myocardial torsion. Even though Err has comparable reproducibility to myocardial torsion there is only modest improvement of reproducibility with repeated analyses based on CoV and no improvement based on ICC. Consequently, one needs to decide whether the positive effects of repeated analyses on intervendor agreement and intravendor reproducibility for most parameters would justify a threefold increased analysis time, especially in the setting of a large volume clinical practice. Doubling or tripling the time of analysis with an increase from about 9–12 to 27–35 minutes may represent a limitation of the feasibility of CMR-FT for clinical routine. From a time versus cost view, the analysis based on three repetitions may consequently not proof cost-effective. From a time versus use standpoint, the clinical applicability of both software types seems comparable because of similar analysis durations for a single case with either software; however the fact that Circle provides built-in torsion measurements within their software interface may well enhance the clinical feasibility of deriving this parameter. In comparison, at present TomTec derived rotational displacement and resulting data need to be further analysed, in the present case with in-house Matlab software, for torsion calculation. An automatic and consistent selection of the apical and basal levels for the estimation of torsion removes the human factor in the selection of sections (in this study not accounted in the results of Circle, the sections were pre-defined in each case). In the present study, reproducibility was comparable using either software suggesting reliability of both approaches.

When interpreting the results of the current study, it is important to note that the main user action involves the manual delineation of the endocardial and epicardial contours in the first frame of an existing sequence of images, to start the tracking algorithm and to correct the initial contours if the tracking is not sufficient or has failed. The identification of these two initial contours is easily and quickly performed by a skilled user. Nevertheless, this factor introduces considerable variability in the results. This can be explained by the intrinsic difficulty to estimate rotation and strain metrics neglecting the out-of-plane movement that the myocardium experiences through the heart cycle. Having two vendors performing similarly in terms of reproducibility suggests that FT in conventional short axis CMR has fundamental limitations that need to be tackled by the combination of different views in an attempt to reconstruct the true three-dimensional (3D) deformations and strains.

### Study limitations

Significantly lower Ecc and resting torsion was found using Circle; however, the sample size of the current study in healthy volunteers is small and needs to be recognised when interpreting the results of the current study. The study did not include patients; however, similar CMR-FT reproducibility between health and disease had been reported before^11^, ^12^, ^13^, ^23^ independent of different patients groups. Consequently, the comparison of different types of CMR-FT software in healthy volunteers is appropriate and the results transferable when studying different disease states.

Furthermore myocardial tagging or speckle tracking echocardiography was not included as an independent reference standard. Notwithstanding this fact, it is important to note that TomTec has been compared to myocardial tagging with excellent agreements^9^ and speckle tracking echocardiography with reasonable to good agreements in the past.^8^, ^24^ Furthermore, the aim of the current study was not to undertake another comparison with myocardial tagging^25^, ^26^, ^27^, ^28^ or speckle tracking echocardiography^8^, ^24^ but simply assess how well the two types of CMR-FT software agree with each other and whether or not both types of CMR-FT software can be used interchangeably.

Global deformation parameters were studied in the present study. Ideally, quantitative tools should be used to derive segmental information in addition to global values. Several studies have shown that segmental analysis does not provide high amounts of reproducibility for myocardial strain within repeated analyses^29^ and repeated studies.^22^ Therefore, the focus was on comparisons of entire sections and global myocardial deformation and rotation. Future refinements for both types of analysis software will possibly allow accurate and reproducible quantification of segmental deformation.

In conclusion, assessment of myocardial strain and torsion is feasible with the two types of commercially available CMR-FT software with reliable detection of increased myocardial deformation with dobutamine stimulation; however, myocardial strain and torsion measurements using both software types are subject to considerable inter and intravendor variability, even when averaging three analysis repetitions. It is important to note that Ecc and resting torsion values obtained from the Circle software are significantly lower as compared to TomTec. For both vendors, Ecc qualifies as the most robust parameter with the lowest variability. Whether or not the widespread availability of CMR-FT software types will allow the methodology to develop into a useful routine clinical tool has yet to be demonstrated.

## Figures and Tables

**Table 1:** Volunteer demographics.

Demographics	Healthy volunteers
Study population, *n*	10
Gender (F/M)	5/5
Age (years)	40.6 (23–51)
LV-EDV (ml/m^2^)	51.0 ± 7.5
LV-ESV (ml/m^2^)	21.7 ± 5.1
LV-EF (%)	57.9 ± 5.6

Continuous variable are expressed as mean ± standard deviation, age is expressed as median (range). Volumetric results have been adopted from.^15^

EDV, end-diastolic volume; ESV, end-systolic volume; CI, cardiac index; EF, ejection fraction.

**Table 2:** Comparison of CMR-FT derived and hemodynamic parameters between rest and dobutamine stimulation.

		Level of dobutamine (μg kg^−1^ min^−1^)			*P* value		
Rest	10	20	Rest vs. 10	Rest vs. 20	10 vs. 20
Tom Tec	Global torsion (°/cm)	2.7 (1.7)	4 (2.1)	4.6 (2.2)	**<0.05**	**<0.01**	0.678
TPK global torsion (ms)	372 (113)	260 (59)	216 (27)	0.13	**<0.01**	**0.05**
Global Ecc (%)	−18.8 (2.9)	−26 (4.2)	−28.1 (3.3)	**<0.01**	**<0.01**	**<0.05**
TPK global Ecc (ms)	337 (27)	228 (47)	179 (17)	**<0.01**	**<0.01**	**<0.05**
Global Err (%)	31.9 (6.3)	40.1 (9.7)	45.6 (4.9)	0.13	**<0.01**	**<0.05**
TPK global Err (ms)	327 (26)	231 (44)	179 (19)	**<0.01**	**<0.01**	0.125

Circle	Global torsion (°/cm)	1.8 (0.9)	3.6 (1.9)	3.6 (1.7)	**<0.05**	**<0.05**	0.515
TPK global torsion (ms)	360 (75)	237 (50)	202 (52)	**<0.01**	**<0.01**	**<0.05**
Global Ecc (%)	−16 (2.7)	−20.7 (2.1)	−21.5 (2.5)	**<0.01**	**<0.01**	0.374
TPK global Ecc (ms)	345 (51)	226 (54)	184 (31)	**<0.01**	**<0.01**	0.051
Global Err (%)	27.6 (6.4)	45.2 (7.1)	49.3 (9)	**<0.01**	**<0.01**	0.139
TPK global Err (ms)	345 (52)	229 (52)	187 (30)	**<0.01**	**<0.01**	0.051

	LV-EDV (ml/m^2^)	51.0 (7.5)	52.7 (9.1)	43.8 (15.4)	0.33	**<0.05**	**<0.01**
	LV-ESV (ml/m^2^)	21.7 (5.1)	14.4 (5.9)	11.4 (4.6)	**<0.01**	**<0.01**	**<0.01**
	LV-SV (ml/m^2^)	29.4 (4.1)	38.3 (7.4)	37.3 (6.5)	**<0.01**	**<0.01**	0.24
	LV-EF (%)	57.9 (5.6)	72.9 (9.5)	77.0 (5.7)	**<0.01**	**<0.01**	**<0.05**
	Mean BP (mmHg)	92 (10)	99 (10)	103 (11)	**<0.01**	**<0.05**	**<0.05**
	Heart rate	69 (10)	85 (17)	113 (12)	**<0.01**	**<0.01**	**<0.01**

Results are reported as mean (SD). Ecc, circumferential LV short axis strain; Err, radial LV short axis strain; TPK, time to peak; ms, milliseconds; BP, blood pressure; Other Abbreviations as in Table 1. Volumetric results have been adopted from.^15^ Bold *p* values indicate a significance level≤0.05.

**Table 3:** Intervendor agreement and reproducibility for torsion, circumferential and radial strain based on the average of three repeated measurements (R3).

		TomTec versus Circle			TomTec			Circle		
Mean Difference (SD)	ICC (95%CI)	CoV (%)	Mean Difference (SD)	ICC (95%CI)	CoV (%)	Mean Difference (SD)	ICC (95%CI)	CoV (%)

Intra-observer	Global torsion (°/cm)	0.77 (1.19)	0.81 (0.63-0.9)	35.08	0.02 (1.06)	0.86 (0.72-0.93)	28.99	0.1 (0.71)	0.88 (0.77-0.94)	22.43
TPK global torsion (ms)	16.05 (105.67)	0.36 (0-0.64)	38.2	4.25 (59.4)	0.78 (0.59-0.89)	21.32	31.32 (72.04)	0.67 (0.41-0.83)	27.12
Global Ecc (%)	4.84 (2.71)	0.81 (0.63-0.91)	12.47	0.09 (0.65)	0.99 (0.99-1.00)	2.69	0.2 (1.25)	0.93 (0.85-0.97)	6.75
TPK global Ecc (ms)	3.73 (20.89)	0.96 (0.92-0.98)	8.28	0.89 (3.5)	1.00 (1.00-1.00)	1.4	1.13 (7.61)	1.00 (0.99-1.00)	3.01
Global Err (%)	1.42 (12.2)	0.32 (0-0.61)	30.71	2.07 (3.95)	0.93 (0.85-0.96)	10.01	0.22 (3.4)	0.96 (0.92-0.98)	8.51
TPK global Err (ms)	8.41 (24.9)	0.94 (0.88-0.97)	9.89	2.05 (14.38)	0.98 (0.95-0.99)	5.8	2.17 (11.03)	0.99 (0.98-1.00)	4.29

Interobserver	Global torsion (°/cm)	0.66 (1.42)	0.84 (0.65-0.92)	42.73	0.12 (0.99)	0.94 (0.87-0.97)	26.83	0.13 (0.98)	0.89 (0.77-0.95)	32.14
TPK global torsion (ms)	7.92 (102.07)	0.55 (0.04-0.79)	37.45	8.13 (58.63)	0.9 (0.78-0.95)	20.89	12.7 (90.99)	0.70 (0.37-0.86)	33.09
Global Ecc (%)	4.82 (3.1)	0.87 (0.72-0.94)	14.3	0.02 (1.06)	0.99 (0.98-1.00)	4.4	0.82 (1.09)	0.97 (0.94-0.99)	5.78
TPK global Ecc (ms)	3.46 (20.42)	0.98 (0.96-0.99)	8.09	0.27 (3.59)	1.00 (1.00-1.00)	1.43	1.69 (8.08)	1.00 (1.00-1.00)	3.19
Global Err (%)	0.11 (13.04)	0.47 (0-0.75)	32.2	1.54 (5.3)	0.92 (0.83-0.96)	13.32	0.39 (3.86)	0.98 (0.95-0.99)	9.59
TPK global Err (ms)	9.21 (31.81)	0.95 (0.9-0.98)	12.65	0.81 (15.74)	0.99 (0.97-0.99)	6.37	2.19 (7.34)	1.00 (1.00-1.00)	2.85

Results are reported as mean (SD).

ICC, intraclass-correlation coefficient; CoV, coefficient of variation; SD, standard deviation; CI, confidence interval. Other abbreviations as in Table 2.

**Table 4:** Intervendor agreement and reproducibility for torsion, circumferential and radial strain based on the average of two repeated measurements (R2).

		TomTec versus Circle			TomTec			Circle		
Mean Difference (SD)	ICC (95%CI)	CoV (%)	Mean Difference (SD)	ICC (95%CI)	CoV (%)	Mean Difference (SD)	ICC (95%CI)	CoV (%)

Intra-observer	Global torsion (°/cm)	0.84 (1.31)	0.78 (0.59-0.89)	38.44	0.21 (1.16)	0.84 (0.68-0.92)	32.19	0.19 (0.78)	0.85 (0.71-0.93)	23.78
TPK global torsion (ms)	23.63 (112.14)	0.30 (0.00-0.60)	40.92	12.91 (69.52)	0.74 (0.52-0.87)	24.75	31.57 (89.32)	0.57 (0.26-0.77)	33.68
Global Ecc (%)	4.77 (2.82)	0.80 (0.61-0.9)	12.97	0.18 (0.84)	0.99 (0.98-0.99)	3.48	0.12 (1.4)	0.91 (0.82-0.96)	7.57
TPK global Ecc (ms)	4.24 (21.97)	0.96 (0.92-0.98)	8.7	1.62 (4.57)	1.00 (1.00-1.00)	1.82	0.56 (5.81)	1.00 (0.99-1.00)	2.29
Global Err (%)	1.51 (12.27)	0.36 (0.00-0.64)	30.64	2.29 (4.35)	0.91 (0.82-0.96)	10.93	0.52 (4.47)	0.93 (0.86-0.97)	11.24
TPK global Err (ms)	12.59 (22.02)	0.96 (0.91-0.98)	8.81	6.07 (19.43)	0.96 (0.91-0.98)	7.83	1.45 (10.83)	0.99 (0.98-1.00)	4.22

Interobserver	Global torsion (°/cm)	0.71 (1.45)	0.84 (0.67-0.93)	43.36	0.13 (0.74)	0.97 (0.94-0.99)	19.67	0.2 (1.06)	0.87 (0.72-0.94)	34.42
TPK global torsion (ms)	12.19 (115.58)	0.50 (0.00-0.77)	43.08	11.44 (65.9)	0.87 (0.72-0.94)	23.53	18.81 (122.07)	0.50 (0.00-0.76)	44.94
Global Ecc (%)	4.72 (3.1)	0.86 (0.71-0.94)	14.31	0.05 (1.04)	0.99 (0.98-1.00)	4.34	0.84 (1.02)	0.98 (0.95-0.99)	5.39
TPK global Ecc (ms)	3.69 (21.82)	0.98 (0.96-0.99)	8.63	0.55 (3.85)	1.00 (1.00-1.00)	1.54	2.04 (7.64)	1.00 (1.00-1.00)	3.01
Global Err (%)	0.08 (12.99)	0.52 (0-0.77)	31.8	1.59 (5.95)	0.90 (0.8-0.96)	14.85	0.77 (4.05)	0.97 (0.94-0.99)	10.02
TPK global Err (ms)	11.13 (31.03)	0.95 (0.9-0.98)	12.38	1.47 (20.05)	0.98 (0.95-0.99)	8.21	1.41 (9.22)	1.00 (0.99-1.00)	3.59

Results are reported as mean (SD).

ICC, intraclass-correlation coefficient; CoV, coefficient of variation; SD, standard deviation; CI, confidence interval. Other abbreviations as in Table 2.

**Table 5:** Intervendor agreement and reproducibility for torsion, circumferential and radial strain based on single measurements (R1).

		TomTec versus Circle			TomTec			Circle		
Mean Difference (SD)	ICC (95%CI)	CoV (%)	Mean Difference (SD)	ICC (95%CI)	CoV (%)	Mean Difference (SD)	ICC (95%CI)	CoV (%)

Intra-observer	Global torsion (°/cm)	0.74 (1.94)	0.68 (0.42–0.83)	54.65	0.02 (1.72)	0.72 (0.48–0.86)	47.01	0.37 (1.54)	0.61 (0.31–0.79)	46.75
TPK global torsion (ms)	30.03 (141.62)	0.02 (0.00–0.38)	51.09	0.19 (96.1)	0.58 (0.27–0.78)	34.31	30.03 (90.75)	0.58 (0.28–0.78)	34
Global Ecc (%)	4.65 (3.01)	0.78 (0.58–0.9)	13.8	0.29 (1.21)	0.97 (0.95–0.99)	5.02	0.29 (2.16)	0.81 (0.63–0.91)	11.7
TPK global Ecc (ms)	3.66 (24.97)	0.95 (0.89–0.98)	9.91	1.44 (5.63)	1.00 (0.99–1.00)	2.24	1.48 (8.58)	0.99 (0.99–1.00)	3.39
Global Err (%)	1.06 (12.75)	0.37 (0.02–0.65)	31.53	4.9 (8.51)	0.75 (0.53–0.87)	20.87	0.25 (4.74)	0.92 (0.84–0.96)	11.99
TPK global Err (ms)	10.02 (20.24)	0.96 (0.92–0.98)	8.1	6.28 (24.86)	0.93 (86–0.97)	10.03	1.61 (17.41)	0.98 (0.95–0.99)	6.76

Interobserver	Global torsion (°/cm)	0.5 (1.6)	0.87 (0.72–0.94)	46.65	0.24 (0.91)	0.97 (0.94–0.99)	23.99	0.07 (1.44)	0.83 (0.64–0.92)	45.85
TPK global torsion (ms)	17.76 (130.12)	0.36 (0.00–0.70)	48	12.28 (96.9)	0.76 (0.49–0.89)	33.87	19.72 (131.03)	0.29 (0.00–0.67)	48.16
Global Ecc (%)	4.96 (3.73)	0.82 (0.62–0.92)	17.2	0.17 (1.09)	0.99 (0.98–1.00)	4.52	1.18 (1.22)	0.97 (0.93–0.99)	6.46
TPK global Ecc (ms)	2.53 (24.73)	0.97 (0.94–0.99)	9.79	1.13 (5.47)	1.00 (1.00–1.00)	2.18	1.6 (9.23)	1.00 (0.99–1.00)	3.65
Global Err (%)	2.27 (14.55)	0.52 (0–0.77)	34.55	3.32 (8.16)	0.87 (0.72–0.94)	19.63	1.56 (5.17)	0.96 (0.91–0.98)	12.85
TPK global Err (ms)	10.16 (37.62)	0.93 (0.85–0.97)	15.06	0.14 (26.5)	0.96 (0.92–0.98)	10.83	3.53 (13.79)	0.99 (0.98–1.00)	5.37

Results are reported as mean (SD). ICC, intraclass-correlation coefficient; CoV, coefficient of variation; SD, standard deviation; CI, confidence interval. Other abbreviations as in Table 2.
